# Diversity Matters for Health: Activity Diversity, Emodiversity, Stressor Diversity, and Happyversity

**DOI:** 10.1093/geroni/igaa057.2247

**Published:** 2020-12-16

**Authors:** Soomi Lee, Emily Urban-Wojcik, David Almeida

## Abstract

Aging theories suggest that diversity of experiences relates to social integration, cognitive reserve, and more psychological resources, all of which are important for successful aging. However, age-related declines may contribute to a monotonous daily life. Emerging studies suggest that activity diversity and positive emotional diversity are associated with better health; yet, we still know little about how a variety of diverse lifestyle indicators are associated with health in adulthood. This symposium showcases contemporary endeavors towards understanding how multiple diversity indicators of adult lifestyle relate to health. Paper 1 examines activity diversity (breadth and evenness of daily activity participation) and tests its associations with overall and nightly sleep health. Paper 2 investigates the relationship between activity diversity and hippocampus volume. Paper 3 examines whether there are age-related differences in the extent to which positive emodiversity attenuates the association between stress and depressive symptoms. Paper 4 examines stressor diversity and how it is associated with blood pressure across age and SES. Paper 5 introduces a novel concept of happyversity (diversity in life satisfaction across different domains) and tests its association with terminal decline. These papers use different project datasets that include different populations of middle-aged and older adults, such as the Midlife in the United States Study, Intraindividual Study of Affect, Health and Interpersonal Behavior, Study of Health and Interactions in the Natural Environment, and German Socioeconomic Panel Study. At the end of these presentations, Dr. Almeida will discuss their theoretical and methodological contributions, and consider challenges and opportunities for future research.

